# The Clinical Effect of Deferoxamine Mesylate on Edema after Intracerebral Hemorrhage

**DOI:** 10.1371/journal.pone.0122371

**Published:** 2015-04-13

**Authors:** Yao Yu, Wei Zhao, Chunpeng Zhu, Zhiping Kong, Yan Xu, Guangzhi Liu, Xuguang Gao

**Affiliations:** 1 Department of Neurology, Peking University People’s Hospital, Beijing, China; 2 Department of Internal medicine, Mancheng country Hospital, Baoding, China; University of Glasgow, UNITED KINGDOM

## Abstract

**Background and Purpose:**

It has been shown that 3 days of 62 mg/kg/day deferoxamine infusion (maximum dose not to exceed 6000 mg/day) is safe and tolerated by intracerebral hemorrhage (ICH) patients. The aim of this study was to investigate the efficacy of deferoxamine mesylate for edema resolution and hematoma absorption after ICH.

**Methods:**

From February 2013 to May 2014, spontaneous ICH patients diagnosed by computed tomography (CT) within 18 hours of onset were evaluated. Patients were randomly divided into two groups: an experimental group and a control group. The treatment of the two groups was similar except that the experimental group received deferoxamine mesylate. Patients were evaluated by CT and neurology scale at the time of admission, and on the fourth, eighth, and fifteenth day (or at discharge) after admission. Patients were followed up for the first 30 days and clinical data of the two groups were compared.

**Results:**

Forty-two patients completed 30 days of follow-up by May 2014; 21 cases in the experimental group and 21 cases in the control group. The control group’s relative edema volume on the fifteenth day (or discharge) was 10.26 ± 17.54, which was higher than the experimental group (1.91 ± 1.94; P < 0.05). The control group’s 1–8 day and 8–15 day relative hematoma absorption were greater than the experimental group (*P* < 0.05).The control group’s relative edema volume on the fourth, eighth, and fifteenth day (or discharge) was higher than the experimental group (*P* < 0.05). Neurological scores between the two groups were not statistically different on the fifteenth day (or discharge) or on the thirtieth day.

**Conclusions:**

Deferoxamine mesylate may slow hematoma absorption and inhibit edema after ICH, although further investigation is required to form definitive conclusions.

**Trial Registration:**

Chinese Clinical Trial Registry ChiCTR-TRC-14004979

## Introduction

Intracerebral hemorrhage (ICH) is a common type of stroke[1,2]. Initial hematoma volume, early hematoma enlargement, secondary hydrocephalus, and perihematoma edema (PHE) are associated with the deterioration of neurological function and poor prognosis[3]. The acute phase of mass effect produced by PHE results in the deterioration of neurological function and seriously affects prognosis[4]. Therefore, identifying PHE and other secondary injury predisposing factors and the implementation of targeted interventions are key steps in reducing mortality, morbidity, and improving prognosis[5]. Currently, there is no therapy for PHE after ICH, aside from surgery and supportive treatment[3–6].

It has been demonstrated that iron ions plays a key role in the formation of PHE, nerve cell death, and tissue damage after ICH[4–7]. Following ICH, hemoglobin and other degradation products, particularly iron released from red blood cells (RBCs), cause brain damage through a variety of mechanisms[8]. Deferoxamine can pass the blood-brain barrier[9], reduce iron accumulation in nerve tissue[10], and has a variety of neuroprotective functions in addition to complexing with iron ions[11]. It has been confirmed that 3 days of 62 mg/kg/day deferoxamine infusion (maximum dose not to exceed 6000 mg/day) is safe and tolerated by ICH patients[6]. Thus, iron chelators are expected to become a new treatment method for ICH[11]. To date, there are but a few studies reporting the clinical efficacy of deferoxamine for PHE and the impact on the hematoma and edema in ICH patients[12,13]. This study is a clinical trial investigating the efficacy of deferoxamine mesylate for hematoma and edema absorption after ICH, and provides clinical evidence for ICH treatment with deferoxamine mesylate.

## Materials and Methods

The protocol for this trial and supporting CONSORT checklist are available as supporting information; see S1 CONSORT Checklist and S1 Protocol.

This study was a single-center, prospective cohort study. From February 1, 2013 to May 1, 2014, patients with spontaneous ICH confirmed by cranial computed tomography (CT) onset within 18 hours were consecutive collected from Mancheng country hospital.

The study protocol was approved by the Mancheng country hospital medical ethics committee, and patients included in this study or their families were informed of the study by researchers and signed informed consent.

### Sample size

The sample size was determined using relative edema volume on the fifteenth day (or discharge) as the main outcome variable. To ensure that the study was designed with a power of at least 0.90, using a significance level of 0.05, the following formula was used to calculate the sample size:

$$\text{N}=[(Z_{\alpha /2}+Z_{\beta }){\sigma /\delta ]}^{2}\;(Q_{1}^{-1}+Q_{2}^{-1})$$

In this case, the *Z*_*α/2*_ is 1.96 and *Z*_*β*_ is 1.282. *Q*_*1*_ and *Q*_*2*_ represent the sample fraction of the two groups. We assumed that the number of patients in the experimental group and the control group were equal, and therefore *Q*_*1*_ and *Q*_*2*_ are both 0.5. We set *σ/δ* as 1. To be able to determine a significant difference in relative edema volume on the fifteenth day (or discharge) between the two groups, it was necessary to include 21 patients in each arm.

### Randomization methods

Randomization was performed using the statistical software SPSS 13.0 (SPSS Inc., Chicago, IL, USA). The two arms were established in a 1:1 ratio, and the random table of the group information was established in the design phase before patient enrolment according to the sequence of the patients enrolled.

### Inclusion criteria

1) Age ≥22 years; 2) spontaneous ICH confirmed by CT; 3) onset within 18 hours; 4) signed informed consent; and 5) clinical status of a stable condition.

### Exclusion criteria

1) Allergic to deferoxamine mesylate and the like; 2) serum creatinine >2 mg/dL; 3) diagnosed with iron deficiency anemia, hemoglobin <7 g/L, or patients requiring transfusion therapy; 4) plans to implement surgical intervention; 5) tumor-related ICH patients, taking warfarin or those with coagulation disorders (INR ≥1.7), ruptured aneurysms, arteriovenous malformations, or venous thrombosis; 6) changes in the midline structure of the brain or patients with herniation; 7) deep coma (Glasgow Coma Scale [GCS] score <5 points); 8) taking iron supplements (≥325 mg of iron) or prochlorperazine; 9) heart failure or need to take vitamin C >500 mg daily; 10) hearing impaired; 11) systolic blood pressure <100 mmHg or diastolic blood pressure <60 mmHg patients, after three consecutive blood pressure measurements; 12) pregnant or lactating women; 13) alcoholism, drug dependence, poor compliance, or other factors that could affect research project completion; 14) the existence of any condition that could increase the patient’s risk; 15) participation in another clinical trial at the same time; or 16) refused to accept cardiopulmonary resuscitation during hospitalization.

### Treatment programs

Patients enrolled in the study were randomly divided into either an experimental group or control group using a random number table by one researcher who was not involved in recruiting patients. The supportive treatment of the experimental group control group is same as the standard therapy, but for the edema, referring the results of studies by Selim et al.[6] and Okauchi et al.[14], we designed a treatment program for the experimental group. Patients in the experimental group received intravenous injection of deferoxamine mesylate 32 mg/kg daily from the first admission day for 3 consecutive days. The infusion rate per hour did not exceed 7.5 mg/kg and the maximum daily dose did not exceed 6000 mg. Patients in the control group did not receive deferoxamine mesylate.

### Data collection

Within 6 hours of admission, patients included in this study or their relatives were investigated using epidemiological research methods, venous blood sample were collected for laboratory examination, imaging data (CT) were recorded, and neurology scale scoring were used to evaluate status.

The modified Rankin score (mRS) and other neurology scale scores were used to evaluate patient status on days 4, 8, and 15 (or discharge). The Bathel index (BI) score was used to evaluate patient status on days 8 and 15 (or discharge). CT imaging was used to evaluate patient hematoma and edema volume using the formula ABC/2 on days 4, 8, and 15 (or discharge).

### Imaging data collection

Patient CT scan data were collected on the day of admission and on days 4, 8, and 15 days (or at discharge). The time of the CT, the ICH site, whether bleeding leaked into ventricles, and other information were recorded. A deputy director of neurology proficient in reading CT data was assigned to calculate hemorrhage volume, absolute edema volume, and relative edema volume. The director calculated patient CT data randomly and was blind to the patient group and other information. Each CT film was evaluated twice, with at least a 2 day interval between evaluations, and averages were calculated.

### Edema and hematoma volume

The formula ABC/2 was used to calculate hematoma volume and hematoma + edema volume. The absolute edema volume = (edema + hematoma) volume—hematoma volume, relative edema volume = absolute edema volume / hematoma volume[15,16]. If hematoma expansion occurred, expansion of hematoma and edema volume was defined as a fraction of the initial hematoma volume and edema volume.

To observe the absorption of the hematoma and to exclude natural hematoma absorption (gradually after 7 days in ICH[17]), we defined the relative absorption of hematoma volume of the first to eighth day: (initial hematoma volume—eighth day hematoma volume) / initial hematoma volume and relative absorption of hematoma volume of the eighth to fifteenth day (or discharge), which equals (eighth day hematoma volume—fifteenth day hematoma volume) / the eighth day hematoma volume.

### Primary and secondary end points

This study aimed to investigate the clinical effect of deferoxamine mesylate on edema after intracerebral hemorrhage. Hematoma volume and edema volume are processes of dynamic change after ICH and, based on the study by Selim et al.,[6] we made the relative edema volume on the fifteenth day (or discharge) as the primary end point. mRS on the fifteenth day (or discharge) and the thirtieth day were used as secondary end points. We defined a mRS score ≥3 as a poor outcome.[18] At the same time, we calculated the relative hematoma absorption from the first to eighth day and the eighth to fifteenth day (or discharge) of the two groups to describe the treatment effects of deferoxamine mesylate. Moreover, we described the mRS, NIHSS, BI, GCS, and GOS scores at admission, and on the fourth, eighth, fifteenth (or discharge), and on the thirtieth day.

### Follow-up data collection

In the first 30 days after admission (±7 days), patients were followed up by telephone or face-to-face interview. mRS and other neurological scales were used to evaluate patients status at this time.

### Blinding method

In this study, the patients were not aware of the detailed information of their treatment and to which group they belonged. The investigator in charge of evaluating the neurological scale and the Deputy Director of Neurology in charge of studying the CT data did not know to which group a patient belonged before exposing the blind. When all the data from the study became available, data according to the group information was renamed group A or group B at random. The data was then given to a statistician who was only aware of these labels, and therefore did not know which group was the experimental or control group before exposing the blind. When the statistician had completed the statistical analysis and reported the results, the group information was completely disclosed. All procedures were supervised by the ethics committee to ensure the blinding method was followed strictly.

### Statistical analysis

All data were processed using statistical software SPSS 13.0 (SPSS Inc., Chicago, IL, USA), and *P* <0.05 was considered statistically significant. The Kolmorogov-Smirnov test was used to test the normality and homogeneity of continuous variables. Repeated measures analysis of variance was used to test the relative absorption of hematoma volume on the first to the eighth day and the eighth to the fifteenth day (or discharge) of the two groups. Repeated measures analysis of variance was used to test the relative absorption of hematoma volume on the first to the eighth day and the eighth to the fifteenth day (or discharge) of the same group. Either the *t*-test or *t*΄-test (when the data were not homogenous) were used to test the relative absorption of hematoma volume on the first to the eighth day of the two groups and the relative absorption of hematoma volume on eighth to the fifteenth day (or discharge) of the two groups. Repeated measures analysis of variance was used to test the relative edema volume on admission, and on the fourth, eighth, and fifteenth day (or discharge) of the two groups. Repeated measures analysis of variance was used to test the relative edema volume on admission, and on the fourth, eighth, and fifteenth day (or discharge) of the same group. The *t*-test or *t*΄-test (when the data were not homogenous) was used to test the relative edema volume at the same time point between the two groups. The Chi-square test was used to compare categorical data between the two groups.

## Results

From February 1^st^, 2013 to May 1^st^, 2014, 106 patients were evaluated by the researchers; 64 patients were excluded from the study, and 42 patients completed the 30 day follow-up. There were 21 patients in the experimental group, and their average age and hematoma volume were 64.2 ± 9.5 years and 15.2 ± 8.8 mL, respectively. Similarly, 21 patients were included in the control group, and their average age and hematoma volume were 60.1 ± 8.7 years and 12.6 ± 10.3 mL, respectively. The baseline data of the 42 patients on admission are shown in Table 1. The CONSORT Flow Diagram is shown in S1 Table.

**Table 1:** Baseline patient demographic characteristics, clinical features, and the main laboratory indicators at admission ($\bar{\text{x}}$ *± s*).

Item	Experimental group	Control group
Sample size	21	21
Age (years)	64.2 ± 9.5	60.1 ± 8.7
Systolic blood pressure (mmHg)	166.7 ± 24.2	178.7 ± 27.6
Diastolic blood pressure (mmHg)	98.5 ± 12.2	102.0 ± 18.6
Laboratory parameters		
FIB (g/L)	2.4 ± 0.5	2.2 ± 0.6
INR	1.1 ± 0.3	1.0 ± 0.1
APTT (S)	24.3 ± 6.4	26.2 ± 3.1
WBC (10^9^/L)	7.1 ± 2.5	7.1 ± 1.7
PLT (10^9^/L)	201.9 ± 54.7	227.5 ± 52.0
GLU (mmol/L)	6.8 ± 1.4	7.0 ± 1.7
CR (μmol/L)	69.5 ± 27.2	68.1 ± 19.4
TG (mmol/L)	1.6 ± 1.0	1.8 ± 1.4
mRS	3.9 ± 0.6	3.8 ± 0.5
NIHSS	9.1 ± 4.6	8.7 ± 5.4
ICH	0.3 ± 0.6	0.6 ± 0.6
GOS	3.0 ± 0.2	3.1 ± 0.3
GCS	13.9 ± 0.7	13.7 ± 1.3
Hematoma volume (mL)	15.2 ± 8.8	12.6 ± 10.3
Occupying volume (mL)	19.9 ± 11.3	16.5 ± 13.1
Absolute edema volume (cm^3^)	4.7 ± 3.9	3.9 ± 4.7
Relative edema volume	0.3 ± 0.2	0.3 ± 0.3

### The influence of deferoxamine mesylate on hematoma absorption after ICH

**Fig 1:**
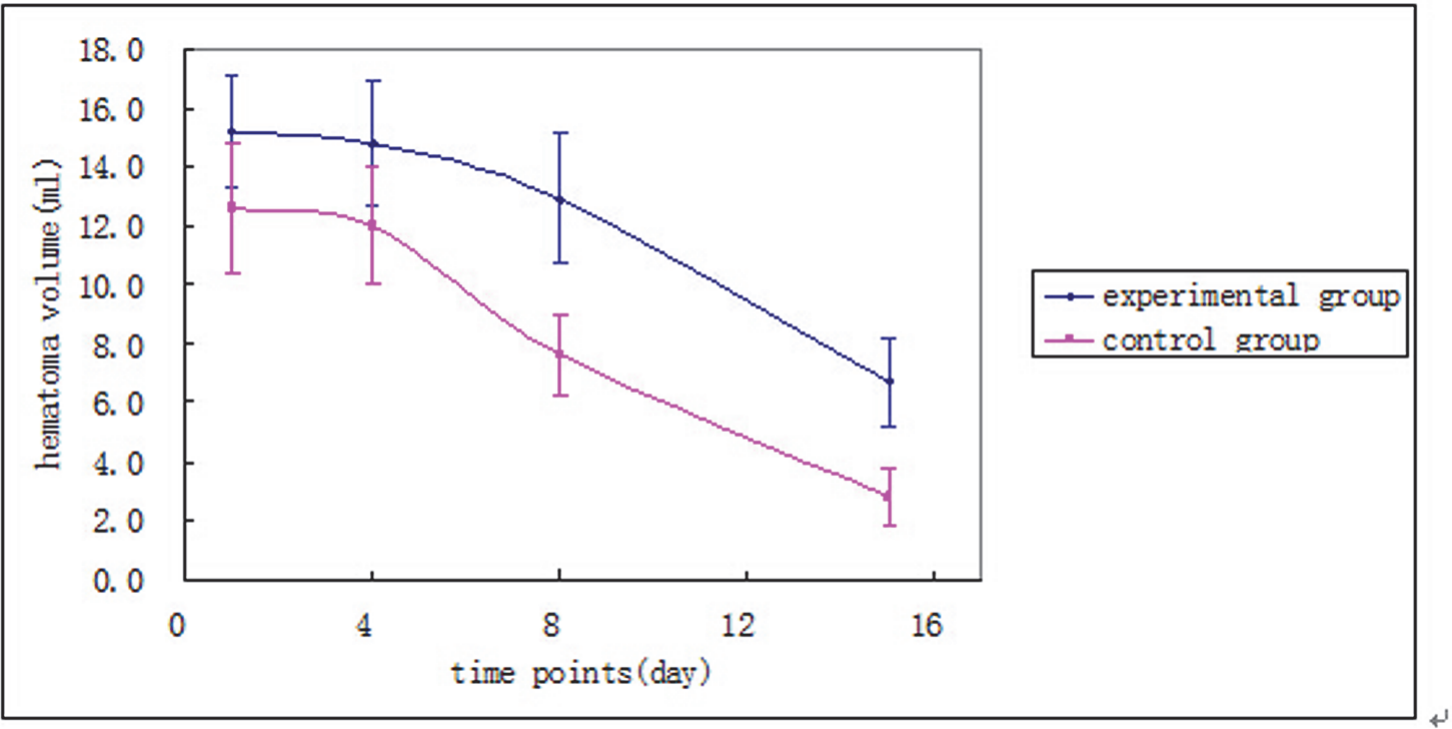
Trends in hematoma volume at admission, and on the 4^th^, 8^th^, and 15^th^ day (or discharge).

Patient average hematoma volume trends in experimental and control groups at the time of admission, and on days 4, 8, and 15 (or discharge) are shown in Fig 1. Relative hematoma absorption of the first to eighth day and the eighth to fifteenth day (or discharge) is shown in Table 2. The difference in the relative absorption of hematoma volume at different time periods was statistically significant by repeated measures analysis of variance (*F* = 53.275, *P* = 0.000); this difference was also significant within groups (*F* = 17.333 and *F* = 48.164, for the experimental and control groups, respectively; *P*-value < 0.05, for both). The difference in the relative absorption of hematoma volume between the two groups was statistically significant according to the repeated measures analysis of variance (*F* = 15.816, *P* = 0.000); the relative hematoma absorption of the control group was higher than the experimental group. The difference in the relative absorption of hematoma volume on the first to the eighth day between the two groups was statistically significant according to the *t*-test (*t* = –2.876, *P* = 0.006). The relative absorption of hematoma volume on the first to the eighth day of the control group was higher than the relative absorption of hematoma volume during the same time period in the experimental group. The difference in the relative absorption of hematoma volume on the eighth to the fifteenth day (or discharge) between the two groups was statistically significant by *t*-test (*t* = –3.077, *P* = 0.004). The relative absorption of hematoma volume on the eighth to the fifteenth day of the control group was higher than the relative absorption of hematoma volume during the same period in the experimental group. There was no interaction between groups and time (*F* = 0.123, *P* = 0.727).

**Table 2:** The relative absorption of hematoma volume on the first to the eighth day and eighth to the fifteenth day (or discharge) of the two groups ($\bar{\text{x}}$ *± s*).

Groups	n	The first to the eighth day	The eighth to the fifteenth day (or discharge)	Sum	*F*	*P*
Experimental group	21	0.21 ± 0. 25	0.55 ± 0.29	0.38 ± 0.32	17.333	0.000
Control group	21	0.41 ± 0.21	0.79 ± 0.21	0.60 ± 0.29	48.164	0.000
sum		0.31 ± 0.25	0.67 ± 0.28	0.49 ± 0.32		
*t* value		-2.876	-3.077			
*P* value		0.006	0.004			

Different group: *F* = 15.816, *P* = 0.000.

Different time: *F* = 53.275, *P* = 0.000 (by Greenhouse-Geisser correction).

Group × time: *F* = 0.123, *P* = 0.727 (by Greenhouse-Geisser correction).

### The influence of deferoxamine mesylate on edema after ICH

**Fig 2:**
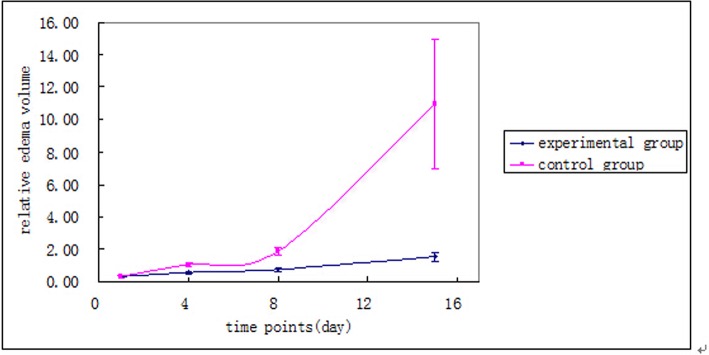
Trends in relative edema volume at admission, and on the 4^th^, 8^th^, and 15^th^ day (or discharge).

Patient average relative edema volume in the experimental group and control group at the time of admission, and day 4, 8, and 15 (or discharge) is shown in Fig 2 and Table 3. The difference in relative edema volume at different time points between groups was statistically significant by repeated measures analysis of variance (*F* = 7.736, *P* = 0.008); which was also the case within groups (*F* = 12.192 and *F* = 5.877 for the experimental and control groups, respectively; *P* < 0.05, for both). The difference in relative edema volume between the two groups was statistically significant by repeated measures analysis of variance (*F* = 6.571, *P* = 0.014); the relative edema volume in the control group was higher than in the experimental group. The difference in relative edema volume on the fourth, eighth, and fifteenth day (or discharge) between the two groups were statistically significant according to the *t*-test or *t*’-test results (*t* or *t*’ values were—3.560, –5.091, and—2.167, respectively; *P* < 0.05, for all). The relative edema volume on the fourth, eighth, and fifteenth day (or discharge) in the control group was higher than the relative edema volume on the fourth, eighth, and fifteenth day (or discharge) in the experimental group. There was an interaction between groups and time (*F* = 4.181, *P* = 0.047).

**Table 3:** The relative edema volume on admission, and the fourth, eighth, and fifteenth day (or discharge) of the two groups ($\bar{\text{x}}$ *± s*).

Groups	n	Admission	The fourth day	The eight day	The fifteenth day (or discharge)	Sum	*F*	*P*
Experimental group	21	0.31 ± 0.23	0.57 ± 0.37	0.68 ± 0.50	1.91 ± 1.94	0.87 ± 1.18	12.192	0.001
Control group	21	0.28 ± 0.29	0.99 ± 0.40	1.96 ± 1.03	10.26 ± 17.54	3.37 ± 9.53	5.877	0.025
sum		0.30 ± 0.26	0.78 ± 0.44	1.32 ± 1.03	6.08 ± 13.03	2.12 ± 6.89		
*t*(*t’*) value		0.354	-3.560	-5.091(t’)	-2.167(t’)			
*P* value		0.725	0.001	0.000	0.042			

Different group: *F* = 6.571, *P* = 0.014.

Different time: *F* = 7.736, *P* = 0.008 (by Greenhouse-Geisser correction).

Group × time: *F* = 4.181, *P* = 0.047 (by Greenhouse-Geisser correction).

### The effects of deferoxamine mesylate on neurological function

#### Comparison of outcome on the fifteenth day

The number of the patients with a mRS ≥3 and <3 in the two groups on the fifteenth day (or at discharge) is shown in S2 Table. There are 11 and 10 patients with a mRS ≥3 in the experimental and control group, respectively; this difference was not statistically significant by Chi-square test (*χ* ^*2*^ = 0.095, *P* = 0.758).

#### Comparison of outcome on the thirtieth day

The number of patients with a mRS ≥3 and <3 in the two groups on the thirtieth day (or at discharge) is shown in S3 Table. There are nine and seven patients with a mRS ≥3 in the experimental and control group, respectively; this difference was not statistically significant by Chi-square test (*χ* ^*2*^ = 0.404, p = 0.525).

Patient average mRS, NIHSS, BI, GCS, and GOS scores and 95% confidence intervals at different time points are shown in S4–S8 Tables. There were no SAEs related to deferoxamine mesylate use in this study.

## Discussion

A large number of free radicals catalyzed by iron are generated and are involved in the formation of edema after ICH. Specifically, a large number of free radicals generated by the direct or indirect iron-mediated damage of the blood-brain barrier via the production of nitric oxide (NO), matrix metalloproteinases (MMPs), and other inflammatory cytokines, which results in cerebral vascular edema[19]. Moreover, the large number of free radicals generated by iron results in cytotoxic cerebral edema through damaging ion transporters on the cell membrane, inducing excitatory amino acid production, intracellular calcium overload and acidosis, and other harmful mechanisms[20]. The mechanism of secondary brain injury after ICH indicates that the intervention of iron overload and range of related damage may be future targets of ICH treatment[21].

The iron chelator deferoxamine is approved by the Food and Drug Administration for the treatment of acute iron poisoning and chronic iron poisoning resulting from long-term blood transfusions and can quickly pass through the blood brain barrier and gather in the brain[22]. Numerous experiments have shown that deferoxamine can chelate iron ions and combine them into stable compounds to prevent further chemical reactions, thereby reducing hematoma and hemoglobin-induced brain edema[23]. Furthermore, deferoxamine is a direct free radical scavenger and can improve tolerance to ischemia-reperfusion injury[24]. Based on these studies, deferoxamine mesylate is a promising new drug for ICH.

In 2011, Selim et al. noted that intravenous infusion of 62 mg/kg/day of deferoxamine mesylate for 3 consecutive days was safe and well tolerated by patients with ICH[6]. The present study is based on the research by Selim et al. and was designed to test the clinical efficacy of deferoxamine mesylate for edema after ICH.

The requirement of onset within 18 hours as inclusion criteria for this study are based on the results of previous studies and animal experiments[6,25]; past experiments have shown that rats with ICH given deferoxamine at 24 hours recover faster than rats with ICH given deferoxamine at 48 hours. Therefore, patients in this study were included and treated when onset was within 18 hours. Although treatment within 18 hours may not be the ideal treatment window, it may be the best time window for the efficacy of deferoxamine for ICH, as a previous study[26] showed that greater peripheral edema extent after ICH is associated with disease progression within 48 hours.

Animal experiments[26] showed that continuous administration of deferoxamine ≥2 days improves the prognosis of rats with ICH, while 7 days is reportedly the best course of treatment. Considering that neurological worsening is highly associated with the rapid growth of edema with in the first 2 to 3 days after ICH[26], hemoglobin dissolved with in the first 2 to 3 days after ICH, and iron ions gathered around the hematoma will be peak at that period[27], we chose the more conservative 3 days as a course of treatment. Based on previous studies[6,28] and the specific constitution of Chinese individuals, we chose 32 mg/kg/day (maximum dose not to exceed 6000 mg/day), which is a moderate dose that is both safe and tolerable in patients with ICH and is most likely to reduce brain edema.

Traditionally, CT is the preferred imaging technique because of perceived superior sensitivity for detecting intracerebral hemorrhage (ICH), better (but by no means ubiquitous) availability, and concerns regarding practicalities related to emergency MRI scanning, so we diagnosed ICH by CT at the admission in this study[29]. In order to observe the effect of deferoxamine for the treatment of brain edema after ICH, patients were divided into either an experimental or control group using a random number table upon admission; the two groups received the same standard treatment, except patients in the experimental group were given deferoxamine.

In this study, we found that the relative hematoma absorption of hematoma on the first to eighth day and the eighth to fifteenth day (or discharge) and the relative absorption of occupying volume on the eight to fifteenth day (or discharge) of the control group was higher than the experimental group, which suggests that deferoxamine mesylate can slow down the absorption of hematoma after ICH, which is accordance with animal studies[30]. Animal experiments have shown that deferoxamine mesylate suppressed ferritin upregulation in the ipsilateral basal ganglia after ICH and hematoma lysis (hematoma volume at day 7: 13.2 ± 4.9 versus 3.8 ± 1.2 mm^3^ in the vehicle-treated group, *P* < 0.01)[30]. However, the mechanisms underlying this relationship require further study. At the same time, we found that the relative edema volume on the fourth, eighth, and fifteenth day (or discharge) were higher than the experimental group, which suggests that deferoxamine mesylate can also slow down the formation of edema after ICH, which is accordance with animal experiments[11,28]. We found that in the process of relative hematoma absorption, there was no interaction between groups and time; the reason for this could be the small sample size, or perhaps that our use of relative hematoma absorption, which could adjust for underlying ICH volume. In the process of evaluating relative edema volume, there was interaction between groups and time, because relative edema volume increases most rapidly in the first 2 days after ICH symptom onset and peaks toward the end of the second week[27]. In our study, time had less of an effect on the experimental group; the reason for this may be the role of deferoxamine, which deserves further study.

In this study, the difference in outcome between the experimental and control groups on the fifteenth and thirtieth days was not statistically significant, which may have been a result of the small sample size. Another reason for this may be that although the extent of the damage and restoration of neurological function is not associated with hematoma and edema volume, but is more related to hematoma and edema site. Therefore, future research needs to explore the effects of deferoxamine mesylate on neurological function in a paired study. We could not conclude that deferoxamine mesylate can accelerate neurologic recovery; larger studies are needed to confirm this hypothesis.

A previous study[31] showed that deferiprone can be effective in attenuating cerebral vasospasm after subarachnoid hemorrhage (SAH) in rabbits. Cabantchik et al.[32] claimed that any chelator applied to regional siderosis, cardiac, neuronal, or endocrine disease ought to preserve both systemic and regional iron levels. The proposed deferiprone-based therapy has provided a paradigm for treating regional types of siderosis without affecting hematological parameters and systemic functions. Devos et al.[33] conducted a clinical trial in which Parkinson’s patients were treated with deferiprone based on the pathophysiological role of iron in Parkinson’s disease (PD). Therefore, future studies should investigate deferiprone as a substitute for deferoxamine.

Current treatment of perihematoma edema is directed at managing raised intracranial pressure (ICP), especially if hydrocephalusis also present[34]. The principles of managing ICP in ICH are borrowed from experience in traumatic brain injury where there is a strong emphasis on maintaining adequate cerebral perfusion pressure according to the status of cerebral autoregulation, although there is much controversy over the use of ICP monitoring at all, let alone by what is the most appropriate location site in the brain[35]. Deferoxamine mesylate is a promising new drug for ICH, although many more studies are required before implementation in clinical practice.

## Limitations

1. The number of sample of this study was small, resulting in fewer positive results; therefore, future studies should expand the sample size.
2. Patients included in this study had small to moderate hematoma volume; therefore, future studies should focus on the effects of deferoxamine mesylate on different hematoma volumes.
3. In this study, patients were enrolled within 18 hours of ICH onset and given intravenous deferoxamine mesylate within 6 hours of admission, which limited the admission of deferoxamine mesylate to within 24 hours of the onset. Therefore, future research should explore the best time window for deferoxamine mesylate administration.
4. Patients in the experimental group were given 32 mg/kg/day (maximum daily dose did not exceed 6000 mg) intravenous deferoxamine mesylate for three consecutive days. Future research should explore the best course of treatment by increasing or decreasing the dose and varying the drug regimen.

## Conclusion

Deferoxamine mesylate may slow the absorption of hematoma and the formation of relative edema volume 15 days after ICH onset. This hypothesis requires further investigation to formulate definitive conclusions.

## Supporting Information

S1 CONSORT Checklist (DOC)

S1 Protocol (PDF)

S1 Table CONSORT 2010 Flow Diagram. (DOC)

S2 Table Modified Rankin Scale score of the two groups on the fifteenth day (or discharge). (DOC)

S3 Table Modified Rankin Scale score of the two groups on the thirtieth day. (DOC)

S4 TableModified Rankin Scale score of the two groups at different time points ($\bar{\text{x}}$ *±s*).(DOC)

S5 TableNIHSS score of the two groups at different time points ($\bar{\text{x}}$ *±s*).(DOC)

S6 TableBathel Index score of the two groups at different time points ($\bar{\text{x}}$ *±s*).(DOC)

S7 TableGlasgow Coma Scale score of the two groups at different time points ($\bar{\text{x}}$ *±s*).(DOC)

S8 TableGOS score of the two groups at different time points ($\bar{\text{x}}$ *±s*).(DOC)
