# Supplementary material for: The Clinical Effect of Deferoxamine Mesylate on Edema after Intracerebral Hemorrhage
Source: PLoS One. 2015 Apr 13;10(4):e0122371. doi: 10.1371/journal.pone.0122371 (PMC4395224; doi:10.1371/journal.pone.0122371)
Supplement: S1 Protocol — (PDF) [file pone.0122371.s002.pdf]

Research Protocol

1. Objective

Investigate the clinical effect of Deferoxamine Mesylate on edema after Intracerebral Hemorrhage.

2. Materials and Methods

This study was a single-center, prospective cohort study. From February 1, 2013 to May 1, 2014, patients with spontaneous ICH confirmed by cranial computed tomography (CT) onset within 18 hours were consecutive collected from Mancheng country hospital. Patients included in this study or their families were informed of the study by researchers and signed informed consent.

Sample size

The sample size was determined using relative edema volume on the fifteenth day (or discharge) as the main outcome variable. To ensure that the study was designed with a power of at least 0.90, using a significance level of 0.05, the following formula was used to calculate the sample size:

$$\mathrm{N} = [(Z_{\alpha/2} + Z_{\beta})\,\sigma/\delta]^2\,(Q_1^{-1} + Q_2^{-1})$$

In this case, the $Z_{\alpha/2}$ is 1.96 and Z_{β} is 1.282. Q_1 and Q_2 represent the sample fraction of the two groups. We assumed that the number of patients in the experimental group and the control group were equal, and therefore Q_1 and Q_2 are both 0.5. We set σ/δ as 1. To be able to determine a significant difference in relative edema volume on the fifteenth day (or discharge) between the two groups, it was necessary to include 21 patients in each arm.

Randomization methods

Randomization was performed using the statistical software SPSS 13.0 (SPSS Inc., Chicago, IL, USA). The two arms were established in a 1:1 ratio, and the random table of the group information was established in the design phase before patient enrolment according to the sequence of the patients enrolled.

Inclusion criteria

1) Age ≥22 years; 2) spontaneous ICH confirmed by CT; 3) onset within 18 hours; 4) signed informed consent; and 5) clinical status of a stable condition.

Exclusion criteria

1) Allergic to deferoxamine mesylate and the like; 2) serum creatinine >2 mg/dL; 3) diagnosed with iron deficiency anemia, hemoglobin <7 g/L, or patients requiring transfusion therapy; 4) plans to implement surgical intervention; 5) tumor-related ICH patients, taking warfarin or those with coagulation disorders (INR ≥1.7), ruptured aneurysms, arteriovenous malformations, or venous thrombosis; 6) changes in the midline structure of the brain or patients with herniation; 7) deep coma (Glasgow Coma Scale [GCS] score <5 points); 8) taking iron supplements (≥325 mg of iron) or prochlorperazine; 9) heart failure or need to take vitamin C >500 mg daily; 10) hearing impaired; 11) systolic blood pressure <100 mmHg or diastolic blood pressure <60 mmHg patients, after three consecutive blood pressure measurements; 12) pregnant or lactating women; 13) alcoholism, drug dependence, poor compliance, or other factors that could affect research project completion; 14) the existence of any condition that could increase the patient's risk; 15) participation in another clinical trial at the same time; or 16) refused to accept cardiopulmonary resuscitation during hospitalization.

Treatment programs

Patients enrolled in the study were randomly divided into either an experimental group or control group using a random number table by one researcher who was not involved in recruiting patients. The supportive treatment of the experimental group control group is same as the standard therapy, but for the edema, referring the results of studies by Selim et al.[1] and Okauchi et al.[2], we designed a treatment program for the experimental group. Patients in the experimental group received intravenous injection of deferoxamine mesylate 32 mg/kg daily from the first admission day for 3 consecutive days. The infusion rate per hour did not exceed 7.5 mg/kg and the maximum daily dose did not exceed 6000 mg. Patients in the control group did not receive deferoxamine mesylate.

Data collection

Within 6 hours of admission, patients included in this study or their relatives were investigated using epidemiological research methods, venous blood sample were collected for laboratory examination, imaging data (CT) were recorded, and neurology scale scoring were used to evaluate status.

The modified Rankin score (mRS) and other neurology scale scores were used to evaluate patient status on days 4, 8, and 15 (or discharge). The Bathel index (BI) score was used to evaluate patient status on days 8 and 15 (or discharge). CT imaging was used to evaluate patient hematoma and edema volume using the formula ABC/2 on days 4, 8, and 15 (or discharge).

Imaging data collection

Patient CT scan data were collected on the day of admission and on days 4, 8, and

15 days (or at discharge). The time of the CT, the ICH site, whether bleeding leaked into ventricles, and other information were recorded. A deputy director of neurology proficient in reading CT data was assigned to calculate hemorrhage volume, absolute edema volume, and relative edema volume. The director calculated patient CT data randomly and was blind to the patient group and other information. Each CT film was evaluated twice, with at least a 2 day interval between evaluations, and averages were calculated.

Edema and hematoma volume

The formula ABC/2 was used to calculate hematoma volume and hematoma + edema volume. The absolute edema volume = (edema + hematoma) volume – hematoma volume, relative edema volume = absolute edema volume / hematoma volume[3,4]. If hematoma expansion occurred, expansion of hematoma and edema volume was defined as a fraction of the initial hematoma volume and edema volume.

To observe the absorption of the hematoma and to exclude natural hematoma absorption (gradually after 7 days in ICH[5]), we defined the relative absorption of hematoma volume of the first to eighth day: (initial hematoma volume – eighth day hematoma volume) / initial hematoma volume and relative absorption of hematoma volume of the eighth to fifteenth day (or discharge), which equals (eighth day hematoma volume – fifteenth day hematoma volume) / the eighth day hematoma volume.

Primary and secondary end points

This study aimed to investigate the clinical effect of deferoxamine mesylate on edema after intracerebral hemorrhage. Hematoma volume and edema volume are processes of dynamic change after ICH and, based on the study by Selim et al.,[6] we made the

relative edema volume on the fifteenth day (or discharge) as the primary end point. mRS on the fifteenth day (or discharge) and the thirtieth day were used as secondary end points. We defined a mRS score ≥3 as a poor outcome.[6] At the same time, we calculated the relative hematoma absorption from the first to eighth day and the eighth to fifteenth day (or discharge) of the two groups to describe the treatment effects of deferoxamine mesylate. Moreover, we described the mRS, NIHSS, BI, GCS, and GOS scores at admission, and on the fourth, eighth, fifteenth (or discharge), and on the thirtieth day.

Follow-up data collection

In the first 30 days after admission (±7 days), patients were followed up by telephone or face-to-face interview. mRS and other neurological scales were used to evaluate patients status at this time.

Blinding method

In this study, the patients were not aware of the detailed information of their treatment and to which group they belonged. The investigator in charge of evaluating the neurological scale and the Deputy Director of Neurology in charge of studying the CT data did not know to which group a patient belonged before exposing the blind. When all the data from the study became available, data according to the group information was renamed group A or group B at random. The data was then given to a statistician who was only aware of these labels, and therefore did not know which group was the experimental or control group before exposing the blind. When the statistician had completed the statistical analysis and reported the results, the group information was completely disclosed. All procedures were supervised by the ethics committee to

ensure the blinding method was followed strictly.

Statistical analysis

All data were processed using statistical software SPSS 13.0 (SPSS Inc., Chicago, IL, USA), and P <0.05 was considered statistically significant. The Kolmorogov-Smirnov test was used to test the normality and homogeneity of continuous variables. Repeated measures analysis of variance was used to test the relative absorption of hematoma volume on the first to the eighth day and the eighth to the fifteenth day (or discharge) of the two groups. Repeated measures analysis of variance was used to test the relative absorption of hematoma volume on the first to the eighth day and the eighth to the fifteenth day (or discharge) of the same group. Either the t-test or t'-test (when the data were not homogenous) were used to test the relative absorption of hematoma volume on the first to the eighth day of the two groups and the relative absorption of hematoma volume on eighth to the fifteenth day (or discharge) of the two groups. Repeated measures analysis of variance was used to test the relative edema volume on admission, and on the fourth, eighth, and fifteenth day (or discharge) of the two groups. Repeated measures analysis of variance was used to test the relative edema volume on admission, and on the fourth, eighth, and fifteenth day (or discharge) of the same group. The t-test or t'-test (when the data were not homogenous) was used to test the relative edema volume at the same time point between the two groups. The Chi-square test was used to compare categorical data between the two groups.

3. Technical Route

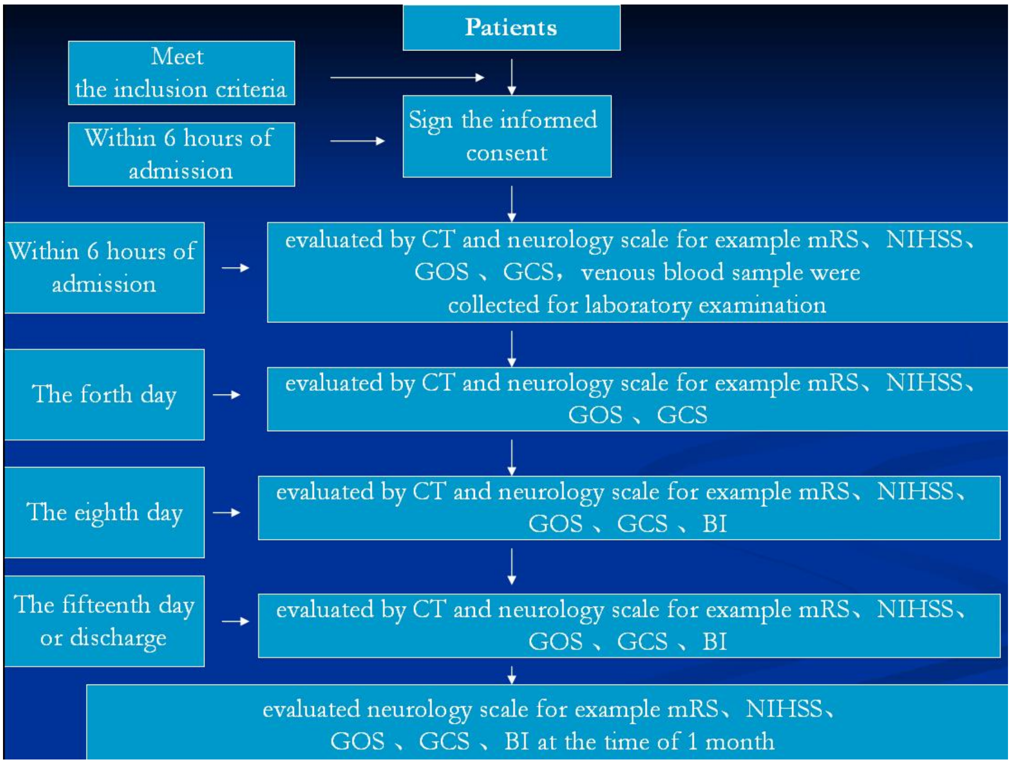

4. **References**

[1]. Selim M, Yeatts S, Goldstein GN, Gomes J, Greenberg S, et al. (2011) Safety and Tolerability of Deferoxamine Mesylate in Patients With Acute Intracerebral Hemorrhage. *Stroke* 42:3067-3074.

[2]. Okauchi M, Hua Y, Keep RF, Morgenstern LB, Xi G. (2009) Effect of deferoxamine on intracerebral hemorrhage-induced injury in aged rats. *Stroke* 40:1858 –1863.

[3]. Freeman WD, Barrett KM, Bestic JM, Meschia JF, Broderick DF, et al. (2008) Computer-Assisted Volumetric Analysis Compared With ABC/2 Method for Assessing Warfarin-Related Intracranial Hemorrhage Volumes[J]. *Neurocritical Care* 9:307-312.

[4]. Gebel JM Jr, Jauch EC, Brott TG, Khoury J, Sauerbeck L, et al. (2002) Natural history of perihematomal edema in patients with hyperacute spontaneous intracerebral hemorrhage. *Stroke* 33:2631–2635.

[5]. Tanaka Y, Marumo T, Shibuta H, Omura T, Yoshida S. (2009)Serum S 100B, brain edema, and hematoma formation in a rat model of collagenase-induced hemorrhagic stroke. Brain Res Bull 78: 158-163.

[6]. Barber M, Roditi G, Stott DJ, Langhorne P.(2004) Poor outcome in primary intracerebral haemorrhage: results of a matched comparison. *Postgrad Med J* 80: 89-92.
