# Supplementary material for: The Clinical Effect of Deferoxamine Mesylate on Edema after Intracerebral Hemorrhage
Source: PLoS One. 2015 Apr 13;10(4):e0122371. doi: 10.1371/journal.pone.0122371 (PMC4395224; doi:10.1371/journal.pone.0122371)
Supplement: S1 Table — (DOC) [file pone.0122371.s003.doc]

**
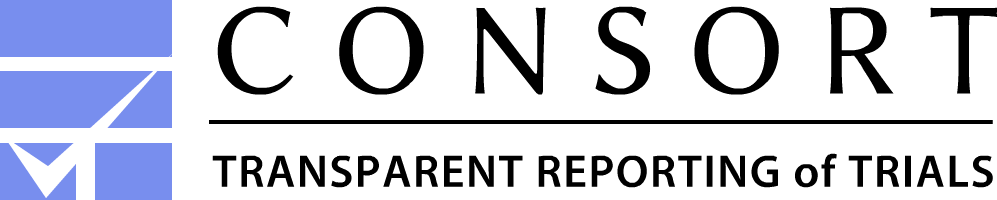
**

**CONSORT 2010 Flow Diagram**

**Allocation**

**Analysis**

**Follow-Up**

**Enrollment**

Assessed for eligibility (n=106 )

Excluded (n= 64 )

♦  Not meeting inclusion criteria (n= 35 )

♦  Declined to participate (n=29 )

♦  Other reasons (n=0 )

Analysed (n= 21 )

Lost to follow-up (give reasons) (n= 21 )

Discontinued intervention (give reasons) (n=0 )

Allocated to intervention (n= 21 )

♦ Received allocated intervention (n=21 )

♦ Did not receive allocated intervention (give reasons) (n= 0 )

Lost to follow-up (give reasons) (n= 21 )

Discontinued intervention (give reasons) (n=0 )

Allocated to intervention (n=21 )

♦ Received allocated intervention (n=21 )

♦ Did not receive allocated intervention (give reasons) (n=0 )

Analysed (n=21 )

Randomized (n=42 )
