# Supplementary material for: The Clinical Effect of Deferoxamine Mesylate on Edema after Intracerebral Hemorrhage
Source: PLoS One. 2015 Apr 13;10(4):e0122371. doi: 10.1371/journal.pone.0122371 (PMC4395224; doi:10.1371/journal.pone.0122371)
Supplement: S3 Table — (DOC) [file pone.0122371.s005.doc]

**Table S2. Modified Rankin Scale score of the two groups on the thirtieth day.**

| Groups | n | Median(range) | Modified Rankin Scale（%） | |
| --- | --- | --- | --- | --- |
|  |  |  | mRS≥3 | mRS＜3 |
| Experimental group | 21 | 2(0-4) | 9（42.9%） | 12（57.1%） |
| Control group | 21 | 1(0-5) | 7（33.3%） | 14（66.7%） |
| *χ^2^* | 0.404 | | | |
| *P* | 0.525 | | | |
