# Supplementary material for: The Clinical Effect of Deferoxamine Mesylate on Edema after Intracerebral Hemorrhage
Source: PLoS One. 2015 Apr 13;10(4):e0122371. doi: 10.1371/journal.pone.0122371 (PMC4395224; doi:10.1371/journal.pone.0122371)
Supplement: S4 Table — (DOC) [file pone.0122371.s006.doc]

**Table S3.** Modified Rankin Scale score of the two groups at different time points

($\bar{\text{x}}$*±s*).

| Groups | Admission  (95% CI) | 4^th^ day  (95% CI) | 8^th^ day  (95% CI) | 15^th^ day (or discharge)  (95% CI) | 30^th^ day (±7days)  (95% CI) |
| --- | --- | --- | --- | --- | --- |
| Experimental group (n=21) | 3.9±0.6  (3.7, 4.1) | 3.7±0.6  (3.4, 4.0) | 3.2±0.7  (3.0, 3.5) | 2.8±1.0  (2.3, 3.2) | 2.0±1.6  (1.4, 2.7) |
| Control group (n=21) | 3.8±0.5  (3.5, 4.0) | 3.6±0.9  (3.2, 3.9) | 3.0±1.2  (2.4, 3.4) | 2.5±1.4  (2.0, 3.1) | 1.9±1.7  (1.2, 2.6) |
