# Supplementary material for: The Clinical Effect of Deferoxamine Mesylate on Edema after Intracerebral Hemorrhage
Source: PLoS One. 2015 Apr 13;10(4):e0122371. doi: 10.1371/journal.pone.0122371 (PMC4395224; doi:10.1371/journal.pone.0122371)
Supplement: S5 Table — (DOC) [file pone.0122371.s007.doc]

**Table S4.** NIHSS score of the two groups at different time points($\bar{\text{x}}$*±s*).

| Groups | Admission  (95% CI) | 4^th^ day  (95% CI) | 8^th^ day  (95% CI) | 15^th^ day (or discharge)  (95% CI) | 30^th^ day (±7days)  (95% CI) |
| --- | --- | --- | --- | --- | --- |
| Experimental group (n=21) | 9.1±4.6  (7.1, 11.0) | 7.8±4.6  (5.9, 9.6) | 4.9±3.7  (3.3, 6.5) | 3.8±3.9  (2.2, 5.6) | 3.2±3.6  (1.9, 4.9) |
| Control group (n=21) | 8.7±5.4  (6.5, 10.9) | 7.2±5.1  (5.1, 9.3) | 4.2±4.1  (2.6, 5.9) | 3.3±4.0  (1.8, 5.0) | 2.8±4.0  (1.3, 4.4) |
