# Supplementary material for: The Clinical Effect of Deferoxamine Mesylate on Edema after Intracerebral Hemorrhage
Source: PLoS One. 2015 Apr 13;10(4):e0122371. doi: 10.1371/journal.pone.0122371 (PMC4395224; doi:10.1371/journal.pone.0122371)
Supplement: S6 Table — (DOC) [file pone.0122371.s008.doc]

**Table S5**. Bathel Index score of the two groups at different time points($\bar{\text{x}}$*±s*).

| Groups | 8^th^ day  (95% CI) | 15^th^ day (or discharge）  (95% CI) | 30^th^ day (±7days)  (95% CI) |
| --- | --- | --- | --- |
| Experimental group (n = 21) | 60.7±32.8  (46.9, 74.8) | 76.2±29.7  (63.1, 88.1) | 79.5±28.1  (66.9, 90.2) |
| Control group (n = 21) | 66.9±31.6  (52.4, 80.5) | 78.1±31.0  (63.8, 90.5) | 80.5±31.9  (66.2, 93.3) |
