# Supplementary material for: The Clinical Effect of Deferoxamine Mesylate on Edema after Intracerebral Hemorrhage
Source: PLoS One. 2015 Apr 13;10(4):e0122371. doi: 10.1371/journal.pone.0122371 (PMC4395224; doi:10.1371/journal.pone.0122371)
Supplement: S7 Table — (DOC) [file pone.0122371.s009.doc]

**Table S6**. Glasgow Coma Scale score of the two groups at different time points

($\bar{\text{x}}$*±s*).

| Group | Admission  (95% CI) | Fourth day  (95% CI) | Eighth day | Fifteenth day (or discharge)  (95% CI) |
| --- | --- | --- | --- | --- |
| Experimental group (n=21) | 13.9±0.7  (13.7, 14.2) | 14.9±0.3  (14.8, 15.0) | 15.0±0.2  (14.9, 15.0) | 15.0±0.0  (15.0, 15.0) |
| Control group (n=21) | 13.7±1.3  (13.1, 14.1) | 14.7±0.7  (14.4, 15.0) | 14.9±0.4  (14.7, 15.0) | 15.0±0.2  (14.9, 15.0) |
