# Supplementary material for: The Clinical Effect of Deferoxamine Mesylate on Edema after Intracerebral Hemorrhage
Source: PLoS One. 2015 Apr 13;10(4):e0122371. doi: 10.1371/journal.pone.0122371 (PMC4395224; doi:10.1371/journal.pone.0122371)
Supplement: S8 Table — (DOC) [file pone.0122371.s010.doc]

**Table S7**. GOS score of the two groups at different time points($\bar{\text{x}}$*±s*).

| Groups | Admission  (95% CI) | Fourth day  (95% CI) | Eighth day  (95% CI) | Fifteenth day (or discharge）  (95% CI) | Thirtieth day (±7 days)  (95% CI) |
| --- | --- | --- | --- | --- | --- |
| Experimental group (n=21) | 3.0±0.2  (3.0, 3.1) | 3.3±0.6  (3.1, 3.6) | 3.8±0.8  (3.5, 4.1) | 4.2±0.9  (3.9, 4.6) | 4.3±0.9  (4.0, 4.7) |
| Control group (n=21) | 3.1±0.3  (3.0, 3.2) | 3.4±0.6  (3.1, 3.7) | 4.0±0.8  (3.7, 4.3) | 4.4±0.9  (4.0, 4.8) | 4.5±0.9  (4.1, 4.8) |
